# Association of Serum Omentin-1, Chemerin, and Leptin with Acute Myocardial Infarction and its Risk Factors

**DOI:** 10.12669/pjms.36.6.2372

**Published:** 2020

**Authors:** Mukhtiar Baig, Kamal Waheeb Alghalayini, Zohair Jamil Gazzaz, Hazem Atta

**Affiliations:** 1 Dr. Mukhtiar Baig, Ph.D. Department of Clinical Biochemistry, Faculty of Medicine, Rabigh, King Abdulaziz University, Jeddah, Saudi Arabia; 2 Dr. Kamal Waheeb Alghalayini, SBIM. Consultant Cardiologist, Department of Medicine, King Abdulaziz University Hospital, Jeddah, Saudi Arabia; 3 Dr. Zohair Jamil Gazzaz, Ph.D. Department of Medicine, Faculty of Medicine, Rabigh, King Abdulaziz University, Jeddah, Saudi Arabia; 4 Dr. Hazem Atta, Ph.D. Department of Clinical Biochemistry, Faculty of Medicine, Rabigh, King Abdulaziz University, Jeddah, Saudi Arabia

**Keywords:** Chemerin, Omentin, Leptin, HbA1c, Myocardial infarction

## Abstract

**Objective:**

To determine the association of serum omentin-1, chemerin, and leptin with acute myocardial infarction (AMI) and its risk factors among individuals admitted with AMI to the coronary care unit (CCU).

**Methods:**

The current case-control study was conducted at the CCU of King Abdulaziz University Hospital (KAUH), Jeddah, Kingdom of Saudi Arabia (KSA), in 2016-2018. A total of 122 AMI patients admitted to CCU, and 52 BMI and age-matched healthy subjects, between 30 and 65 years of age, were included.

**Results:**

Chemerin and omentin-1 are independent predictors of the incidence of MI. Furthermore, serum omentin-1 was significantly lowered while chemerin and hsCRP levels were found to be significantly raised among the individuals with AMI compared to the healthy subjects, and no notable change was found in the serum leptin level. Serum omentin-1, chemerin, and leptin were significantly correlated with weight, BMI, waist circumference in patients, and control subjects. Binary logistic regression analysis displayed that the occurrence of MI is positively correlated with fasting plasma glucose (FPG), TC, TG, LDL-C, hsCRP, and chemerin and in a negative manner with HDL-C, and omentin. The chemerin and omentin-1 were also linked with the MI in multiple logistic regression analysis.

**Conclusions:**

The present results indicated that the serum omentin levels were significantly lowered while chemerin and hsCRP levels were found to be markedly raised among patients. No change was found in serum leptin levels. Serum chemerin and omentin-1 levels were independently associated with the MI. It appears that these parameters may be used to assess the risk spectrum of CAD.

## INTRODUCTION

Globally, cardiovascular diseases (CVDs) are among the top few causes of death of millions of individuals. Controlling the risk factors such as lifestyle modification, glycemic control, BP, and BMI as they have a more significant impact on the CVDs may prevent the CVDs. If this is ignored, and no preventive steps were taken in this regard, then the mortality due to CVDs may increase to 23.3 million by 2030.^1^

Omentin-1 is mainly a useful adipocyte-derived adipokine, and its lower levels are found in patients of carotid atherosclerosis and CAD,^2^,^3^ which indicates its cardioprotective role. Chemerin is an adipokine produced from mature adipocytes.^4^ It is a pro-inflammatory cytokine, and its levels in serum are found to be raised in individuals with obesity and cardiovascular diseases and thus has shown an association with insulin resistance, metabolic disorders, and CVDs.^5^-^7^ Leptin is a small peptide produced by adipocytes and was found to be related with coronary artery disease (CAD), atherosclerosis, endothelial dysfunction, and MI.^8^,^9^ Leptin is considered a pro-inflammatory adipokine and its level is associated with the metabolic risk factors.^10^

Literature indicates that omentin-1 has protective and chemerin, and leptin have a damaging role in atherosclerosis^2^-^4^, but still, there is inconsistency in their role in the AMI. Moreover, these parameters have not been discussed much in AMI patients in KSA, so there is a scarcity of available data about these parameters’ involvement in MI in our local population. Therefore, this study is intended to explore the association of serum omentin-1, chemerin, and leptin levels with risk factors of AMI and the association of the three adipokines with AMI among individuals admitted to the CCU of KAUH, Jeddah, KSA.

## METHODS

The current case-control study was conducted at the CCU of KAUH, Jeddah, KSA, in 2016-2018. The data were collected from 122 AMI patients admitted to the hospital between 30 and 65 years of age, and they fulfilled the study inclusion criteria.

The ethical research committee of the Faculty of Medicine, KAU, Jeddah, approved the research (Ref No. 183-15), and the data was collected after taken written consent from all the subjects or their attendants. Patients having other comorbidities like valvular heart disease, any malignant disease, other inflammatory diseases, infectious disease, septicemia, blood-related disorders, collagen disorders, taking steroids, liver, diabetes mellitus and kidney diseases were excluded from the research. Fifty-two control healthy subjects with similar age and BMI were inducted from the university’s faculty members and non-teaching staff. Detailed medication history was taken from cases and controls. If any subject in the control group was taking hypoglycemic, hypolipidemic and antihypertensive medication or any other medication regularly, those were excluded. We included only those patients that first diagnosed to have an MI. A generalized questionnaire was designed to acquire data regarding the gender, age, weight, height, waist and hip circumferences, lifestyle patterns, educational background, and others. The majority of our study population was Saudi, followed by Indians, Sudani, Pakistani, Syrians, and Egyptians.

Five ml blood was taken in the morning on the day one of their admission in the CCU, and serum was separated and stored at -80°C for analysis. The lipid profile, HbA_1c_, and FPG levels were measured by an autoanalyzer. The human ELISA kits were used to measure omentin-1, chemerin, leptin, and hsCRP and supplied by the Beijing Mesochem Technology Co., Ltd, Beijing, China.

The data has been analyzed using SPSS version 23. The correlation of omentin-1, chemerin, and leptin with different variables was measured by Pearson correlation. Initially, a simple logistic regression analysis was applied to determine the association of study variables with MI, and then multiple stepwise logistic regression analysis was computed. The p-value < 0.05 was considered significant.

## RESULTS

There were 105 males (age 54.38±9.00) and 17 females (age 58.41±9.03 yrs) among cases while 34 males (age 53.41±6.26 yrs) and 18 females (age 53.33±5.62 yrs) in control subjects. The lipid profile was considerably deranged in the diseased group compared to the control counterparts. FPG and HbA1c were significantly higher in patients. Besides this, serum omentin-1 was significantly lowered while chemerin and hsCRP levels were found to be markedly raised among patients (Table-I).

**Table-I:** Comparison of basic characteristics of myocardial infarction patients and healthy control subjects.

Parameters	Control N=52	Cases N=122	P-value	95% CI of the difference	
	
	MEAN (SD)	MEAN (SD)		Lower	Upper
Age (Yrs)	53.38 ±5.99	54.94 ±9.08	0.18	-.75	3.87
Males	53.41±6.26	54.38±9.00	0.487	-1.79	3.73
Females	53.33±5.62	58.41±9.03	0.053	-.06	10.22
Waist Circumf (cm)	90.79±12.49	98.12±15.41	.001*	.24	8.40
W/H Ratio	0.93±0.20	0.97±.36	.45	-.06	.15
BMI (Kg/m^2^)	27.68±4.35	27.93 ±5.22	-1.38	1.87	-1.27
BP Systolic (mm Hg)	123.65±11.40	125.89±18.	0.33	-2.27	6.73
BP Diastolic (mm Hg)	78.56±6.78	76.20±12.20	.11	-5.23	.51
FPG (mmol/L)	5.05±.31	6.22±1.78	.001*	.83	1.50
HbA1c (Gm%)	4.95±.39	6.61±1.69	.001*	1.34	1.98
TC (mmol/l)	4.05±.81	4.56 ±1.21	.002*	.19	.81
TG (mmol/l )	1.40±.62	2.07±.84	.001*	.44	.89
HDL (mmol/l)	1.16±.34	.88±.21	.001*	-.38	-.18
LDL (mmol/l)	2.85±.72	3.30±.99	.004*	.15	.75
Omentin-1 (ng/ml)	246.57±60.82	177.80±65.09	<.001*	-89.64	-47.90
Chemerin (ng/ml)	99.45 ±57.68	173.80 ±60.78	.001*	54.77	93.92
Leptin (ng/ml)	8.68±2.43	8.33±2.51	.39	-1.16	.46
hs-CRP (mg/L)	.96±1.24	6.12±2.17	.001*	4.64	5.68

“BMI= Body mass index, FPG=Fasting plasma glucose, HBA1c=Hemoglobin A1C,

TC= Total cholesterol, LDL-C= Low-density, lipoprotein cholesterol,

HDL-C = High-density lipoprotein cholesterol, hsCRP=high-sensitivity C-reactive protein.”

P-value <0.05 is considered as significant,

* statistically significant

Serum omentin-1, chemerin, and leptin were significantly linked with body weight, BMI, waist circumference in patients, and control subjects. Serum omentin-1 level had no significant correlation with TC, TG, HDL-C, and LDL-C, while serum chemerin had a significant positive relationship with TC, TG, and HDL-C, and serum leptin had a positive relationship with TC, and HDL-C. Serum omentin-1 had a negative correlation with chemerin, leptin, and hsCRP, while chemerin exhibited a positive correlation with leptin and hsCRP in patients and control group. Serum leptin had a positive connection with hsCRP in patients and the control group (Table-II).

**Table-II:** Correlation of serum omentin, chemerin, and leptin levels with baseline clinical, and biochemical characteristics.

Variables	Omentin-1 (ng/ml)		Chemerin (ng/ml)		Leptin (ng/ml)	

	Controls	Cases	Controls	Cases	Controls	Cases

	r (P-value)	r (P-value)	r (P-value)	r (P-value)	r (P-value)	r (P-value)
Age (years)	.03(.79)	-.01(.85)	-.02(.91)	.05(.54)	-.07(.61)	.10(.26)
Height (meters)	-.09(.52)	-.07(.42)	-.02(.88)	.05(.56)	.10(.47)	.09(.28)
Weight (Kg)	-.56(<.001)	-.47(<.001)	.51(<.001)	.60(<.001)	.63(<.001)	.52(<.001)
BMI (kg/m2)	-.56(<.001)	-.60(<.001)	.67(<.001)	.70(<.001)	.73(<.001)	.71(<.001)
Waist (cm)	-.34(.01)	-.54(<.001)	.52(<.001)	.62(<.001)	.45(.001)	.64(<.001)
Hip circum(cm)	-.23(.09)	-.27(.003)	.19(.16)	.28(.002)	.29(.03)	.32(<.001)
W/H Ratio	-.02(.86)	-.19(.03)	.29(.03)	.22(.02)	.06(.63)	.22(.02)
FPG (mmol/L)	-.01(.97)	-.15(.09)	.19(.17)	.19(.03)	.07(.58)	.19(.03)
HBA1c (%)	.08(.54)	-.21(.02)	.01(.96)	.20(.03)	-.12(.40)	.17(.06)
TC (mmol/L)	.09(.54)	-.07(.47)	-.08(.55)	.18(.04)	.10(.46)	.18(.04)
TAG (mmol/L)	-.09(.54)	-.06(.54)	.07(.63)	.19(.04)	-.05(.71)	.00(.96)
HDL-C (mmol/L)	-.14(.32)	-.17(.06)	-.04(.75)	.31(<.001)	.03(.80)	.33(<.001)
LDL-C (mmol/L)	.05(.71)	.004(.96)	.12(.42)	.09(.27)	.21(.13)	.11(.22)
hsCRP (mg/L)	-.19(.17)	-.23(.009)	.19(.16)	.85(<.001)	.08(.54)	.48(<.001)
Omentin-1 (ng/ml)	1	1	-.44(.001)	-.42(<.001)	-.43(.001)	-.42(<.001)
Chemerin (ng/ml)	-.44(.001)	-.42(<.001)	1	1	.57(<.001)	.64(<.001)
Leptin (ng/ml)	-.43(.001)	-.42(<.001)	.57(.001)	.64(<.001)	1	1

The incidence of MI is positively associated with blood sugar, TC, TG, LDL-C, hsCRP, and chemerin and a negative connection with HDL-C, and omentin-1 (Table-III).

**Table-III:** Simple logistic regression analysis with MI.

Variable	β	SE	Wald χ2	p-value	OR	95% CI
Age	0.023	0.020	1.285	0.257	1.023	0.983 – 1.065
BMI	0.010	0.034	0.089	0.765	1.010	0.946 – 1.079
W/H Ratio	0.715	1.047	0.466	0.495	2.044	0.262 – 15.92
FPG	2.763	0.621	19.784	0.000	15.84	4.69 – 53.54
TC	0.499	0.169	7.106	0.008	1.567	1.126 – 2.181
TAG	1.393	0.306	20.72	0.000	4.027	2.211 – 7.338
HDL-C	-5.416	1.022	28.093	0.000	0.004	0.001 – 0.033
LDL-C	0.569	0.202	7.969	0.005	1.766	1.190 – 2.622
hsCRP	1.416	0.231	37.68	0.000	4.122	2.622 – 6.478
Omentin-1	-0.016	0.003	28.672	0.000	0.984	0.979 – 0.990
Chemerin	0.023	0.004	30.91	0.000	1.024	1.015 – 1.032
LEP	-0.057	0.067	0.728	0.393	0.945	0.829 – 1.077

In multiple logistic regression analysis, chemerin, and omentin-1 were also independently associated with the MI (Table-IV).

**Table-IV:** Multivariate logistic regression analysis with MI.

Variable	β	SE	Wald χ2	p-value	OR	95% CI
Chemerin	0.019	0.008	27.41	0.000	1.020	1.011 – 1.058
Omentin-1	-0.009	0.004	14.548	0.000	0.991	0.984 – 0.997

## DISCUSSION

Chemerin and omentin-1 appear to be independent predictors of MI. Patients with acute AMI showed a concurrent increase in serum chemerin and hsCRP, along with a significant decrease in omentin levels. Serum omentin-1 is significantly negatively linked with weight, BMI, waist circumference, W/H ratio, HbA_1C_, hsCRP, chemerin and leptin in MI patients while no significant association with TC, TG, LDL-C, and HDL-C was found. Our results are similar to many studies that found lower omentin-1 levels in CAD subjects, and it was negatively linked with BMI and waist circumference and independently associated with CAD.^3^,^11^,^12^

Serum omentin-1 level has no significant relationship with cholesterol, TAG, HDL-C, and LDL-C, in both groups, and a few other studies also found no correlation.^12^,^13^ It is proposed that obesity negatively controls omentin expression and release into the circulation.^12^ However, there was no difference in the BMI of the present study subjects, so it excludes any influence of body weight, BMI, or obesity. A few possible ways of involvement of omentin-1 in the MI process have been mentioned in the literature.^14^,^15^ Omentin- 1 upsurges insulin sensitivity, and insulin resistance is reflected as an imperative threat for subclinical atherosclerosis.^14^ A recent study proposed a defending role of omentin against endothelial dysfunction, and they described it directly supports endothelial role among subjects with diabetes and CVD risk factors.^15^

Serum chemerin level is an independent predictor of MI risk, and it was substantially higher in AMI patients compared to control subjects. Moreover, it has a significant positive link with TC, TG, and HDL-C, hsCRP, blood sugar, and HbA1c. Serum chemerin had a positive correlation with anthropometric parameters in patients and control subjects. Our findings are in accordance with other studies.^16^-^18^ This again suggests that the role of chemerin in the pathological process of MI as it showed the association with lipid profile and inflammatory markers in these patients. The association should be explored further by longitudinal studies in this area.

The present study results demonstrated that chemerin concentrations had a positive correlation with TC, TG, and negative with HDL-C, and these all are atherosclerotic risk factors. This relationship shows an unwavering link between chemerin levels and atherosclerosis progression in MI patients. The results of the present research are in harmony with several other reports in patients with atherosclerosis.^11^,^19^

However, there have been conflicting results showing chemerin is not associated with CVD in diabetic patients but correlated significantly with important elements of metabolic disease.^20^ The present study results denoted that chemerin levels were connected with hs-CRP and TG levels in patients with MI, and this association was statistically significant. This shows that inflammatory markers like hs-CRP and chemerin levels are associated with CVD and may be used to assess the risk spectrum.

Several mechanisms have been proposed regarding the involvement of chemerin to the development of atherosclerosis such as chemerin being a chemokine stimulates the macrophages and dendritic cells migration towards tissue injury sites^21^ and macrophages develop foam cells, that produce many inflammatory mediators, which cause vascular smooth muscle cell movement and production^22^ and rises the production of reactive oxygen species in these cells.^23^

Leptin is considered a pro-inflammatory adipokine, and its level is usually increased in CAD. Notably, our study displayed a positive relationship of serum leptin levels with BMI, waist circumference, FPG, TC, HDLC, chemerin, and hsCRP and negatively with omentin in MI patients. Most of these factors are considered metabolic risk factors. The present study results are more or less similar to another study.^10^ Studies have shown that serum leptin and hsCRP were significantly correlated in cardiac patients, and patients with CAD show higher serum leptin levels.^9^ In contrast to the present results, a recent study demonstrated substantially higher serum leptin concentrations in ACS patients than control subjects. However, they also reported serum leptin was positively correlated with BMI, hypertension, and female gender in the patients.^24^

In literature, variable results were found regarding the association of serum leptin with lipid parameters.^10^,^25^ Similar to our findings, a meta-analysis stated that raised leptin levels are not correlated with stroke and CVD risks.^26^ Hence the role of serum leptin as a risk factor in AMI patients and other CVDs is debatable and needs more longitudinal studies to develop the causal association. In a nutshell, the correlation between these adipokines shows that there is crosstalk among these in MI patients, but at present, their interplay is unidentified.

### Limitations of the study

The first limitation, the degree of atherosclerosis, was not compared with that of the biochemical markers. Secondly, since it was a cross-sectional study, therefore, a robust causal relationship could not be developed in this case. In some of the patients’ FPG and HBA1c level was that of prediabetic level, and it could be one of the confounding factors in our results.

## CONCLUSION

It is indicated that the serum omentin levels were significantly lowered while chemerin and hsCRP levels were found to be markedly raised among patients. No change was found in serum leptin levels. Serum chemerin and omentin-1 levels were independently associated with the MI. It appears that these parameters may be used to assess the risk spectrum of CAD. Further research is needed to determine the association of these markers with the degree of atherosclerosis and infarction.

### Authors’ Contribution:

**MB:** Conceived the idea, did practical work, drafted manuscript and responsible and accountable for the accuracy or integrity of the work.

**KWG, ZJG, HA:** Collected data and reviewed and edited manuscript.
